# Tenofovir vaginal film as a potential MPT product against HIV-1 and HSV-2 acquisition: formulation development and preclinical assessment in non-human primates

**DOI:** 10.3389/frph.2023.1217835

**Published:** 2023-08-10

**Authors:** Sravan Kumar Patel, Hrushikesh Agashe, Dorothy L. Patton, Yvonne Sweeney, May A. Beamer, Craig W. Hendrix, Sharon L. Hillier, Lisa C. Rohan

**Affiliations:** ^1^ Department of Pharmaceutical Sciences, School of Pharmacy, University of Pittsburgh, Pittsburgh, PA, United States; ^2^ Magee-Womens Research Institute, Pittsburgh, PA, United States; ^3^ Department of Obstetrics and Gynecology, University of Washington, Seattle, WA, United States; ^4^ Department of Medicine, Johns Hopkins University School of Medicine, Baltimore, MD, United States; ^5^ Department of Obstetrics, Gynecology, and Reproductive Sciences, University of Pittsburgh, PA, United States

**Keywords:** HIV prevention, genital herpes, multipurpose technology, tenofovir, vaginal film, women health

## Abstract

Tenofovir (TFV) is an adenosine nucleotide analog with activity against HIV and HSV-2. Secondary analyses of clinical trials evaluating TFV gel as pre-exposure prophylaxis (PrEP) for HIV have shown that gel formulations of TFV provide significant protection against both HIV and HSV-2 acquisition in women who had evidence of use. An alternate quick-dissolving polymeric thin film, to deliver TFV (20 and 40 mg) has been developed as a potential multipurpose technology (MPT) platform. Film formulation was developed based on excipient compatibility, stability, and ability to incorporate TFV doses. Placebo, low dose (20 mg), and high dose (40 mg) films were utilized in these studies. The developed film platform efficiently incorporated the high dose of TFV (40 mg/film), released more than 50% of drug in 15 min with no *in vitro* toxicity. Pharmacological activity was confirmed in an *ex vivo* HIV-1 challenge study, which showed a reduction in HIV-1 infection with TFV films. Films were stable at both doses for at least 2 years. These films were found to be safe in macaques with repeated exposure for 2 weeks as evidenced by minimal perturbation to tissues, microbiome, neutrophil influx, and pH. Macaque sized TFV film (11.2 mg) evaluated in a pigtail macaque model showed higher vaginal tissue concentrations of TFV and active TFV diphosphate compared to a 15 mg TFV loaded gel. These studies confirm that TFV films are stable, safe and efficiently deliver the drug in cervicovaginal compartments supporting their further clinical development.

## 1. Introduction

Approximately 38.4 million people are currently (2021) living with human immunodeficiency virus (HIV), which causes acquired immunodeficiency syndrome (AIDS) (1). An additional 40.1 million people have died of AIDS-related causes since the start of the HIV pandemic. Women, especially those 15–24 years old, are at increased risk of infection compared to men in the same age bracket. In sub-Saharan Africa (SSA), 6 in 7 new infections in the 15–19 age group are among adolescent girls and young women (1). While progress on vaccine development continues, to date no HIV vaccine is available. Therefore, the need to provide alternative prevention strategies such as topical pre-exposure prophylactic (PrEP) products for female use is paramount. Infection with herpes simplex virus-2 (HSV-2), which causes genital herpes, significantly increases the risk of HIV acquisition. Past infection by HSV-2, the leading cause of genital ulcers, is noted in more than one-third of the general population in parts of SSA (2). As per the World Health Organization, an estimated 491 million people worldwide aged 15–49 years have HSV infection (3). Women are twice as likely to contract HSV-2 infection compared to men, which is due to the efficient sexual transmission from men to women and relevant sociocultural factors. The high prevalence of HSV-2 in population vulnerable to HIV reinforces the need for combined prevention of HIV and HSV-2 using multipurpose prevention technologies (MPTs).

Topical PrEP includes products that are applied vaginally or rectally to prevent transmission of HIV and other sexually transmitted infections (STIs). Several studies have explored such products for prevention of HIV-1 acquisition (4, 5) and combined HIV-1 and HSV-2 acquisition (6). The most advanced of these products is the dapivirine intravaginal ring, which showed reduced HIV-1 infection in women in two large Phase III clinical studies (ASPIRE and the RING Study) (7, 8) and is currently recommended for use by women at substantial risk of HIV by the World Health Organization (WHO). PrEP products evaluated to date have been investigated for either coitally-dependent (on-demand) or independent (long-term) use. On-demand products provide an option for protection, which is easily reversible and provides flexibility of dosing without the risk of long-term exposure to drugs (4).

The current work provides rational development and safety testing of a tenofovir (TFV) vaginal film dosage form as an on-demand multipurpose technology (MPT) product against acquisition of HIV and HSV-2. TFV is an adenosine nucleotide analog with activity against HIV and HSV-2 (9). TFV salt forms are widely used in oral products for HIV treatment (10). TFV has been formulated into hydrogels, nanoparticles, nanoparticles-in-films, nanofibers, oral and vaginal tablets, films, and intravaginal rings (11–17). In multiple preclinical and clinical evaluations of PrEP, TFV has been shown to reduce HIV acquisition (18). Daily use of a combination oral tablet containing tenofovir disoproxil fumarate (a TFV salt form) and emtricitabine (Truvada®) was shown to protect from HIV acquisition (10, 19). Three clinical trials evaluated a TFV vaginal gel for PrEP (10, 18, 20, 21), however, inconsistent efficacy results were observed, which were partly attributed to low user adherence leading to reduced protection (22). Interestingly, a separate post-hoc analysis of one of the trials (CAPRISA 004) showed that TFV gel use reduced HSV-2 acquisition by 51% compared to placebo in women that adhered to the TFV gel product (23). Efficacy of vaginally delivered TFV in preclinical models of HSV-2 acquisition has been well established (24). Anti HSV-2 effect of oral TFV prodrugs and combination products has also been investigated in several studies with modest to no effect on HSV-2 acquisition (25, 26). High local fluid and tissue levels of TFV and TFV-diphosphate (TFV-DP), the active metabolite of TFV, will likely improve efficacy.

Some of the known problems with gels such as messiness, leakiness, and non-stealth characteristics could negatively impact user adherence, although these claims have not been proven. Nevertheless, alternate dosage forms with improved user acceptability are advantageous. Polymeric thin films have been identified as an acceptable dosage form option by users for vaginal applications in several studies (27, 28). Films are low cost, applicator-free products that can be used in a discreet manner if required. Moreover, due to their small size with low mass (<0.5 g), films are less likely to cause any undesirable effect on innate antimicrobial factors such as microbiome and glycome (29). Given these advantages, vaginal film formulations containing TFV were developed as an alternative delivery system to gel formulations.

Previous clinical studies, which evaluated the TFV gel product, utilized TFV at 1% w/v delivered in a 4 ml volume (equivalent to 40 mg TFV per dose). The goal of this work was to develop a stable and safe film dosage form that can incorporate TFV at levels equivalent to that previously used. Incorporating TFV in a vaginal film platform was met with several challenges related to physical instability. In this work, systematic and rational formulation development of TFV vaginal films as well as preclinical safety and pharmacokinetic (PK) assessment in pigtail macaques is presented. The developed platform has been evaluated in women (published elsewhere) and found to be safe (30, 31).

## 2. Materials and methods

### 2.1. Materials

Film excipients included sodium carboxymethylcellulose low viscosity (NaCMC-LV; Spectrum Chemicals, New Brunswick, NJ, USA), hydroxypropyl methylcellulose (HMCE5; Methocel E5 Premium LV and K4M, DOW chemicals, Midland, MI, USA), hydroxyethyl cellulose (HEC; Natrosol 2,50l Pharm, Ashland Polymers, Wilmington, DE, USA), and polyvinyl pyrrolidone K-90 (PVPK90; Fluka, St. Louis, MO, USA). CellTiterGlo™ assay kit was obtained from Promega, Madison, WI, USA. All the other chemicals and reagents were purchased from Fisher Scientific and Spectrum Chemicals.

### 2.2. Formulation development

#### 2.2.1. Drug-polymer compatibility using microscopy

Physical stability of TFV was assessed in a series of polymers such as HPMCE5, HEC, PVPK90, and NaCMC-LV. TFV was dissolved in MilliQ water using equimolar sodium hydroxide on a magnetic stirrer. Polymers were then added into TFV containing solution and mixed thoroughly to achieve polymer: drug ratio of 1:1, 2:1, 3:1, 4:1, 5:1 and 6:1. The resulting solution (0.5 ml) was transferred onto a 12-well cell culture plate and dried at 65°C for 3.5 h. The culture plate was then stored over saturated salt solution of 75% relative humidity (RH) at 40°C. Presence of crystals was examined under a microscope on day 7.

#### 2.2.2. Film manufacturing

Films were manufactured using solvent cast method. A liquid blend containing all excipients was prepared by weighing required amounts of excipients and mixing in MilliQ water using an overhead mixer (Eurostar power control visc, IKA, Wilmington, NC, USA) at 50 rpm to achieve complete polymer dissolution. Glycerin was used as a plasticizer. TFV containing polymer blend was prepared by dissolving TFV in the polymer mix. The final pH was adjusted to 6–6.5 using equimolar sodium hydroxide for TFV containing formulations. To manufacture films, the polymer solution was poured on an automatic film applicator (4,340, Elcometer, MI, USA) and dried at 71°C for 16 min. The polymer sheet was peeled off the applicator and cut into 2″ × 2″ unit doses using a die cutter press (Tipmann Die Cutter, IN, USA). TFV was loaded in films at 1% or 2% w/w in the formulation mix, which produced 20- (low dose) or 40-mg (high dose) per unit dose of a 2″ × 2″ film. Films utilized in non-human primate (NHP) studies were cut into 1.1″ × 1.1″ to accommodate anatomical differences between humans and pigtail macaques.

### 2.3. Characterization of TFV films

#### 2.3.1. Physical properties

Weight and thickness of the films were measured using a calibrated balance and calipers respectively. Water content was determined using Karl Fischer autotitrator (890 Titrando, Metrohm, FL, USA). A TX-XT Plus texture analyzer (TA instruments, DE, USA) was used for mechanical characterization. To determine puncture strength, films were placed on the film holder and a puncture probe (spherical end: 1/8 inch diameter) was passed mechanically at 1 mm/sec through the center of the film holder's aperture. The puncture strength was calculated using the following formula:

(1) $$\mathrm{Puncture}\;\mathrm{strength}\;\left(\frac{N}{\mathrm{mm}}\right)\;=\;\frac{\mathrm{Force}\;\mathrm{at}\;\mathrm{break}\;\mathrm{point}\;(N)}{\mathrm{Thickness}\;\mathrm{of}\;\mathrm{the}\;\mathrm{film}\;(\mathrm{mm})}$$

#### 2.3.2. Drug content in films

TFV content in individual films was determined using solid phase extraction (SPE) of TFV and subsequent quantitation by ultra-performance liquid chromatography (UPLC, Acquity, Waters Corporation, MA, USA) equipped with a TUV detector and Empower data acquisition and processing software. For SPE, TFV-containing film was dissolved in 40 ml milliQ water. One ml of the film solution was further diluted with equal volume of 2% formic acid. One ml of this diluted solution was loaded on a previously activated SPE cartridge [Oasis MCX extraction cartridge, 1 cc (30 mg), Waters, USA]. The residual polymer was washed by eluting the SPE cartridge with 1 ml of 2% formic acid. TFV was extracted with 5% methanolic ammonium hydroxide solution from the eluent. Extracted TFV was estimated after an appropriate dilution with MilliQ water using UPLC. TFV was detected at 260 nm. Separation was achieved by injecting 3 μl of solution on an Acquity UPLC BEH C18 column (1.7 µm 2.1 × 50 mm, Waters) at ambient temperature. The flow rate was maintained at 0.3 ml/min. The mobile phase consisted of 90% phosphate buffer (10 mm K_2_HPO_4_ and 4 mm t-Butylammonium bisulfate) adjusted to pH 5.7, and 10% methanol. Drug content was estimated based on a linear regression equation generated from calibration standards.

#### 2.3.3. *In vitro* release

*In vitro* release of TFV from the films was assessed using a USP 4 flow-through apparatus (CE7 smart, Sotax Corporation, MA, USA) connected to a fraction collector. TFV films were placed into 12 mm polycarbonate cells. Dissolution was carried out by circulating 100 ml of 1× PBS at a flow rate of 162 ml/min for 1 h in a closed loop configuration at 37°C. At pre-determined time points, 0.5 ml samples were collected using a programmable fraction collector. Samples were analyzed for TFV amount by UPLC as described above after appropriate dilutions with MilliQ water.

#### 2.3.4. Compatibility with lactobacilli

Film compatibility with *Lactobacillus* species was assessed by standard microbicide safety test (32) using two American Type Culture Collection strains *L. crispatus* 33,197 and *L. jensenii* 25,258 and one clinical strain of *L. jensenii*, LBP 28Ab. TFV films were dissolved in 1.25 ml ACES [N- (2-Acetamido)-2-aminoethanesulfonic acid] buffer. This solution was mixed with 1.25 ml of lactobacilli suspension in 1× phosphate buffered saline (pH 7.4). The bacterial suspension containing dissolved film was incubated at 37°C for 30 min. Samples were taken before and after incubation was complete. Bacterial viability was determined by standard plate count. A sample was considered compatible with lactobacilli if the reduction in viability was <1 log10. ACES buffer treated or untreated bacterial suspension served as controls for the experiments.

#### 2.3.5. Toxicity in TZM-bl cells

*In vitro* toxicity of TFV film towards TZM-bl cells was evaluated by standard CellTiter-Glo® assay. TFV containing and placebo films were dissolved in 4 ml of saline. Ten-fold serial dilutions were made up to 1:10^7^ of original solution. TZM-bl cells were plated at 1 × 10^4^/well in a 96-well clear view plate and left to adhere overnight. Cells were treated with dilutions of film solution and incubated for 48 h at 5% CO_2_ and 37°C. After 48 h, half the media was replaced with CellTiter-Glo® and luminescence was recorded. Percent viability was compared against untreated cells that received cell culture media and incubated similarly.

#### 2.3.6. *Ex vivo* anti-HIV activity and toxicity

Human ectocervical tissues (explants) from pre-menopausal women (IRB # PRO09110431) were pre-exposed to TFV film containing solutions and experiments were conducted as previously reported (33, 34). Briefly, tissues were secured in a transwell plate with the epithelial side up. Tissues in culture were exposed to control (no treatment), placebo, or 20 or 40 mg TFV films dissolved in 2 ml media. Each transwell received 100 µl of treatment media followed by 100 µl of HIV-1_BaL_ (5 × 10^4^ TCID_50_) in media. After 24 h culture, explants were washed, fresh media was added and incubated at 37°C/5% CO_2_. Media collected on various days for 21 days (and replenished) was assayed for p24 to assess infectivity. Ectocervical tissue toxicity was also conducted after 24 h exposure to treatments or control (200 µl volume) in 12 well plates. After exposure, explants were washed by dispensing DPBS. The explants were transferred and incubated with MTT solution at 37°C/5% CO_2_ for 3 h and optical density (OD) of the solution was recorded at 595 nm. The explants were collected, dried overnight, and weight was recorded to correct OD by dividing with tissue weight.

#### 2.3.7. Stability testing

Films manufactured at two doses were subjected to a 24-month long-term stability at 25°C/60% RH and a 6-month accelerated stability at 40°C/75% RH according to International Conference on Harmonisation (ICH) guidelines. Stability samples collected at different time points were assessed for weight, thickness, TFV content, water content, puncture strength, and dissolution. Additionally, compatibility of films with lactobacilli was evaluated at specific time points.

### 2.4. Tenofivir film assessment in macaques

#### 2.4.1. Test products

For testing film product PK and safety in macaques, the size of the films used was 1.1″ × 1.1″, which is approximately one-third of the human size 2″ × 2″. Films containing 11.2 mg and 5.1 mg TFV (equivalent to 40 and 20 mg/film human dose respectively) and a drug-free placebo film (matched formulation with no active drug) were evaluated in the multiple dosing safety study. Of note, pharmacokinetic evaluation was performed using the high dose 11.2 mg film (equivalent to 40 mg human size film) and compared against a gel product (15 mg dose).

#### 2.4.2. Animals

Sexually mature female *Macaca nemestrina* were obtained from a colony of animals at the Washington National Primate Research Center. Prior approval for use of the monkeys in the protocols was obtained from the Institutional Animal Care and Use Committee at the University of Washington. Animals were handled humanely, and experiments were performed within the National Institutes of Health's laboratory animal use guidelines. Animals were not hormonally synchronized or otherwise altered to control for menstrual or hormone status.

#### 2.4.3. Tenofovir film safety and PK evaluation in pigtail macaques

Films were evaluated in the NHP model for PK and safety after vaginal administration. Six macaques were enrolled in the multiple dosing safety study. Three test articles including two TFV films (5.1 mg/film and 11.2 mg/film) and a placebo film were evaluated. A three-arm crossover study design was utilized, where each animal controlled for herself by completing each of three arms of the study. A minimum of three-week recovery period was incorporated between experiments. TFV or placebo film products were administered daily to the vaginal fornix on days 1–5 and 8–11 (Table 1). After a 30-minute resting period, biological samples were collected. Samples were also collected on follow-up days 12 and 15. Cervicovaginal colposcopy, pH and cytology smears, and vaginal swabs for microflora were collected prior to film insertion and at 30 min after product application. Complete sampling schedule is shown in Table 1.

**Table 1:** Sampling schedule for safety assessment. Sample collection was made before (0) and 30 min after (30) film placement.

Assessment	Time (min)																			
	Day 1		Day 2		Day 3		Day 4		Day 5		Day 8		Day 9		Day 10		Day 11		Follow-up	
	0	30	0	30	0	30	0	30	0	30	0	30	0	30	0	30	0	30	Day 12	Day 15
Film administration	x		x		x		x		x		x		x		x		x			
Vaginal swabs	x	x	x	x	x	x	x	x	x	x	x	x	x	x	x	x	x	x	x	x
Colposcopy	x		x				x		x		x				x				x	x

Standardized colposcopic assessments were conducted by a team of three cross-trained technologists following WHO Guidelines (35) and standardized colposcopy guide designed specifically for pigtail macaque studies (36). Vaginal and ectocervical mucosal surfaces were evaluated for erythema, edema and epithelial integrity as well as any unusual findings. Observations were noted on daily examination record forms and documented by digital photography. Vaginal secretion samples were collected with polyester tipped swabs. Vaginal swabs were utilized for microbiota, vaginal pH and cytology assessments.

Comparative PK was evaluated in a separate experiment, after a single vaginal administration of a 1.1″ × 1.1″ 11.2 mg TFV film or 15 mg TFV gel (1.5 ml of 1% TFV gel), in eight animals per arm. Blood samples collected at baseline, 1, 2, 4, 6, 24, 48, and 168 h were processed for plasma and stored frozen until analysis. Vaginal biopsies were collected at 24 h and 168 h after dosing. Plasma and tissue concentrations of TFV and tissue levels of TFV-diphosphate (TFV-DP) were quantified using validated analytical methods. Non-compartmental PK parameters were estimated (Phoenix WinNonlin v.8.3; Certara, Cary, NC).

#### 2.4.4. Identification of key microbiota by cultivation

Vaginal swabs were inoculated onto agar plates for semi-quantitative culture analysis. Inoculum on each plate was streaked into four quadrants to isolate colonies. Columbia agar with 5% sheep blood (PML Microbiologicals, Wilsonville, OR) and BBL™ Human bi-layer Tween agar (HBT; Becton Dickinson and Company, Sparks, MD) plates were incubated aerobically in 5%–6% CO_2_, 36–37°C, for at least 48 h and used to isolate and identify the following microorganisms: *Lactobacillus* species, viridans *Streptococcus*, beta-hemolytic (Group A–D, F, or G) *Streptococcus*, *Enterococcus* species, *Escherichia coli*, aerobic indole-negative Gram-negative rods, *Staphylococcus aureus*, coagulase-negative staphylococci, diphtheroids, yeast, and other aerobic Gram-positive rods and cocci. Laked Blood Kanamycin agar (PML Microbiologicals), HBT, and DifcoTM Rogosa Selective Lactobacillus agar (prepared on-site; Becton Dickinson and Company) plates were incubated anaerobically, 36–37°C, for 4–7 days and used to identify Lactobacillus, non-pigmented and pigmented anaerobic gram-negative rods. Identification of microorganisms was done using colony and bacterial morphologies and phenotypic tests described in Manual of Clinical Microbiology (37). This semi-quantitative analysis was previously described and correlated to quantitative log growth in colony forming units per milliliter vaginal fluid (cfu/ml) (38). Growth within the initial zone of inoculum, 1+, was equivalent to 10^2^ cfu/ml; the second quadrant, 2+, was 10^5^ cfu/ml; the third quadrant, 3+, was 10^6^ cfu/ml; and the fourth quadrant, 4+, was 10^7^ cfu/ml. Lactobacillus and viridans Streptococcus were additionally tested for the production of hydrogen peroxide using tetramethylbenzidine agar plates, prepared in-house (38, 39).

### 2.5. Statistics

Data was evaluated for statistical significance using one-way and two-way analysis of variance (ANOVA) with Tukey's post-hoc tests where applicable. PK parameters were tested for paired differences among formulations (Friedman test) and, if statistically significant, between each pair of formulations (Wilcoxon signed ranks test; IBM SPSS, v.25.0. Armonk, NY). A difference of *p* < 0.05 was considered statistically significant. To analyze film stability data for drug content, linear regression and 95% confidence interval bands were used.

## 3. Results

### 3.1. Formulation development and characterization of TFV films

Thin film dosage forms intended for vaginal use possess small size and mass (<0.5 g). Therefore, incorporating large doses of drug in polymeric thin films can be challenging. Drug solubility in the film matrix plays an important role in the film quality as it affects the film's visual aspects as well as drug release and storage stability. For TFV film development, our efforts were centered around formulating a stable film platform, wherein TFV exists in a solubilized form and remains molecularly dispersed under storage and use.

#### 3.1.1. Excipient selection and formulation development

TFV has pH-dependent solubility with optimum solubility observed at pH 6.5. It was identified that with the use of sodium hydroxide and by increasing the polymer ratio, TFV can be solubilized, and crystallization inhibited. In preformulation studies, short-term storage (7 days) under an accelerated condition (40°C/75% RH) served as a screening method to identify excipients that efficiently incorporated TFV without crystallization. Several film-forming polymers (HPMCE5, HEC, PVPK90 and NaCMC-LV) were evaluated for their ability to achieve high drug loading level of TFV. Supplementary Figure S1 shows microscopy images of TFV mixed with various polymers at 1:1, 2:1, 3:1, 4:1, 5:1 or 6:1 polymer to TFV ratio (P:T). At a P:T ratio that was equal to or greater than 3, no crystal could be detected after 7 days in samples containing HEC, PVPK90 or NaCMC-LV. The samples retained their transparency as well, indicating at least micron-scale miscibility between TFV and polymer. For HEC containing samples, banded spherulites were observed at P:T 1:1, while crystals grew into much looser fibers at P:T 2:1. For PVPK90 containing samples, banded spherulites were observed at P:T 1:1 and P:T 2:1. For NaCMC-LV containing samples, crystals appeared to be much more irregular. Notably HPMCE5 was found to be highly non-uniform and clear phase separation was observed. At low P:T ratios namely 1:1, 2:1 or 3:1, separation occurred throughout the samples while at high P:T ratios namely 4:1, 5:1 or 6:1, separation occurred on the edges. Based on these results, both HEC and PVPK90 were found to be suitable polymers to incorporate TFV.

To increase viscosity which supports manufacturability during the film coating phase, NaCMC-LV was incorporated. The formulations were divided into groups containing either HEC or PVPK90. Increasing NaCMC-LV increased viscosity in both groups (Supplementary Table S1). A 2% w/w NaCMC-LV was found to be optimal for manufacturability. To improve film quality such as flexibility, well-known film forming polymer, HPMCE5, was included in the matrix (HEC or PVPK90 based). Even though HPMCE5 and NaCMC-LV when tested individually at 2% w/w were unable to impart any advantage to TFV solubilization, the final film formulation remained crystal-free as evidenced from x-ray diffraction pattern of the mixture (Supplementary Figure S2). Microscopy was not conclusive on these samples due to their translucent nature. Glycerin was incorporated as a humectant and plasticizer to improve the tactile properties. The final formulation selected was HEC-based because the films formed were superior and easy to detach from the substrate. The final optimum formulation contained HEC, NaCMC-LV, HPMC E5, and glycerin in the ratio shown in Table 2. Formulations containing TFV at 1% w/w (20 mg/2″ × 2″ film) and 2% w/w (40 mg/2″ × 2″ film) utilized the same composition of excipients.

**Table 2:** TFV formulations showing percent (% w/w) of each ingredient.

Ingredient	Dose	
	20 mg/film	40 mg/film
HEC	6	6
HPMC E5	6	6
NaCMC-LV	2	2
Glycerin	2	2
Sodium Hydroxide	0.14	0.28
Tenofovir	1	2
MilliQ Water	82.86	81.72

#### 3.1.2. Film characterization and stability testing

**Figure 1:**
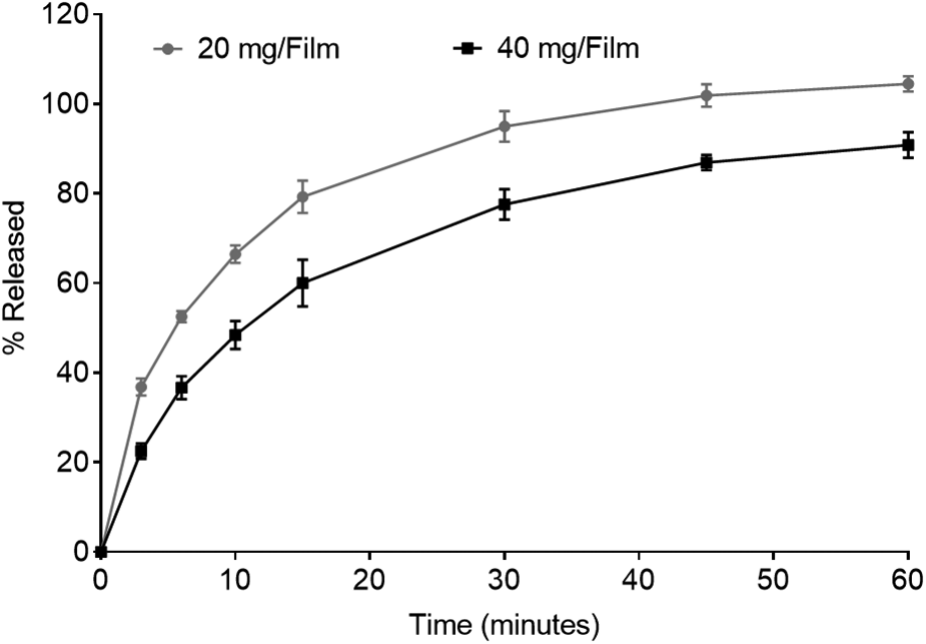
*In vitro* dissolution profile showing drug release from 20 mg and 40 mg tenofovir films for 60 min (*n* = 3–4).

**Figure 2:**
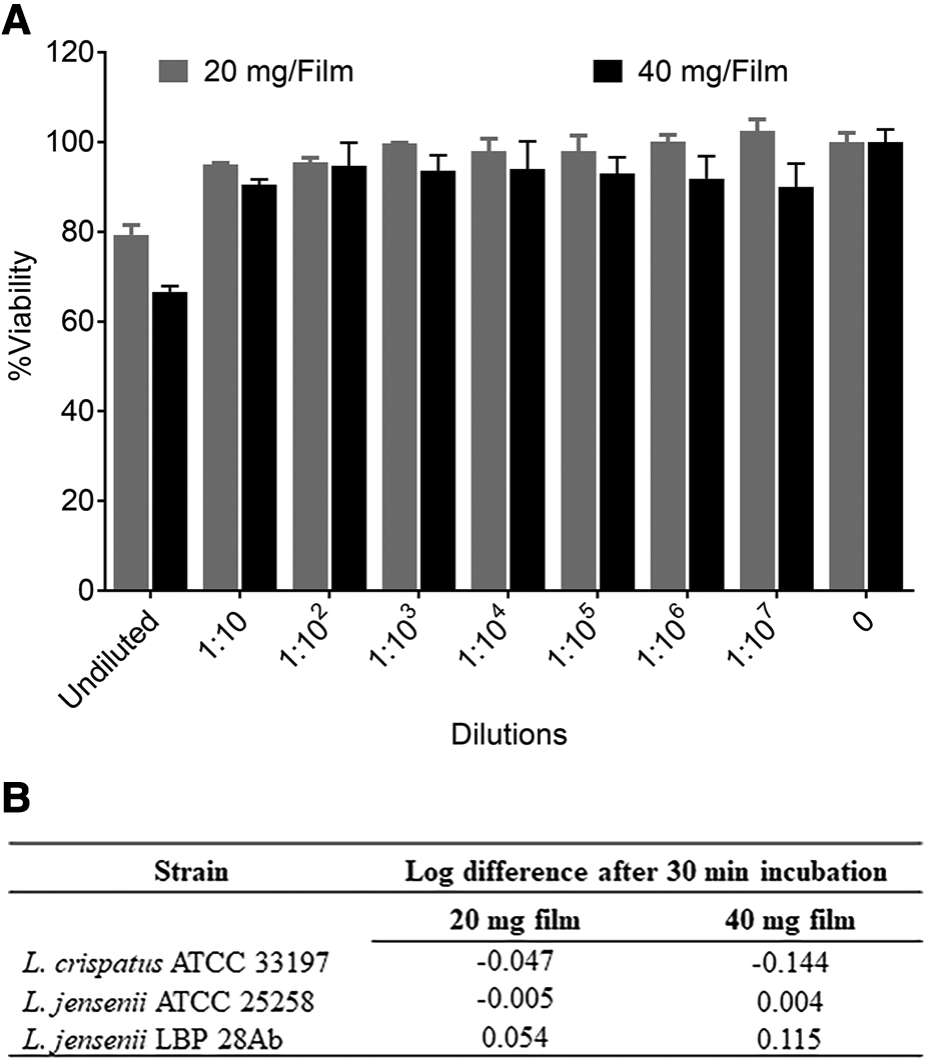
*In vitro* safety of tenofovir containing films. (**A**) Percent viability (mean ± standard deviation) of TZM-bl cells exposed to different dilutions of film solution (*n* = 3) ranging from 0.5 pg/ml to 5 mg/ml TFV (low dose film) and 1 pg/ml to 10 mg/ml (high dose film) compared to unexposed cells (*n* = 6). (**B**) Toxicity of films to lactobacilli at single concentration (8 and 16 mg/ml for low and high dose films respectively).

**Figure 3:**
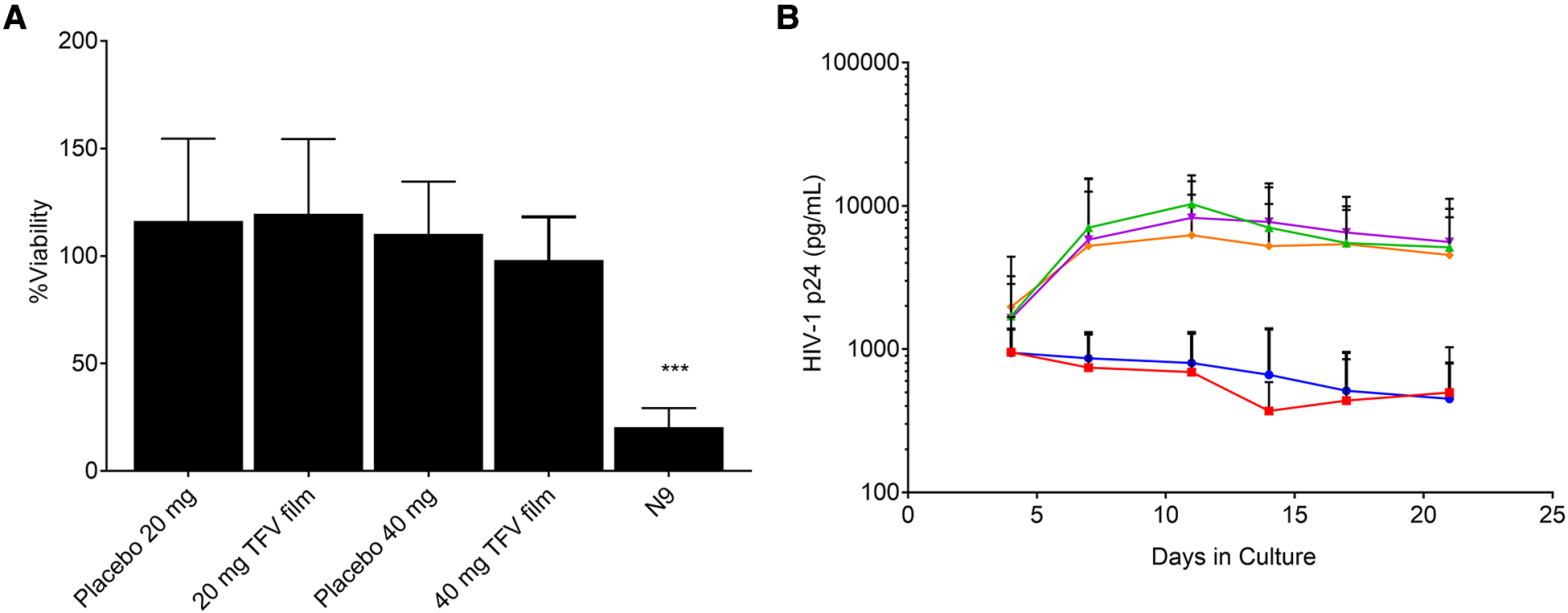
*Ex vivo* evaluation of films in ectocervical tissues (**A**) tissue viability (*n* = 5–6) and (**B**) Protection from infectivity in an *ex vivo* HIV-1 challenge model (*n* = 5–6). Orange = control, magenta = placebo film (20 mg), green = placebo film (40 mg), red = 20 mg tenofovir film, blue = 40 mg tenofovir film.

The characterization of low and high dose TFV films is shown in Table 3. TFV films were smooth, soft, flexible and translucent in nature. Drug loading in films was determined to be within 85%–115% of the target dose levels. As shown in Figure 1, an immediate drug release was observed with greater than 50% of TFV released within 15 min, which reached plateau in 60 min. Visually, TFV films showed complete solubilization in aqueous media. Water content (<10% w/w) remained within the acceptance criteria. *In vitro* toxicity assessment ensured that TFV films do not affect the viability of TZM-bl cells and lactobacilli strains (Figure 2). TFV and placebo films exhibited minimal impact on ectocervical tissue viability (Figure 3A) compared to the positive control N-9 (*p* < 0.001). In the explant HIV-1_BaL_ challenge study, placebo films showed increase in infectivity with time and remained comparable to control group (Figure 3B). Both 20 mg and 40 mg TFV films showed reduced HIV-1 p24 at all days tested, confirming that TFV from films can protect tissues from HIV. A statistically significant (*p* < 0.05) difference was observed between TFV-containing groups compared to placebo and control at all the time points tested. Both low and high dose films showed similar *ex vivo* anti-HIV activity. Overall, the TFV films had acceptable characteristics including dissolution, safety, and anti-HIV activity.

**Table 3:** Day zero characterization of TFV films (2″ × 2″).

Property	20 mg/film	40 mg/film
Appearance	Soft, flexible, translucent	Soft, flexible, translucent
Weight (mg)	362.90 ± 15.96	390.65 ± 20.83
Thickness (µm)	103.64 ± 20.63	110.91 ± 5.39
Water content (%)	3.49 ± 0.51	8.15 ± 0.71
Puncture strength (N/mm)	67.78 ± 10.57	51.49 ± 3.32
Drug content (mg/film)	19.74 ± 0.46	42.42 ± 1.99
Drug release (% at 15 min)	77.72 ± 9.07	60.04 ± 5.18
Lacto toxicity	Not toxic	Not toxic

The data is presented as mean ± SD (*n* = 3–11).

**Figure 4:**
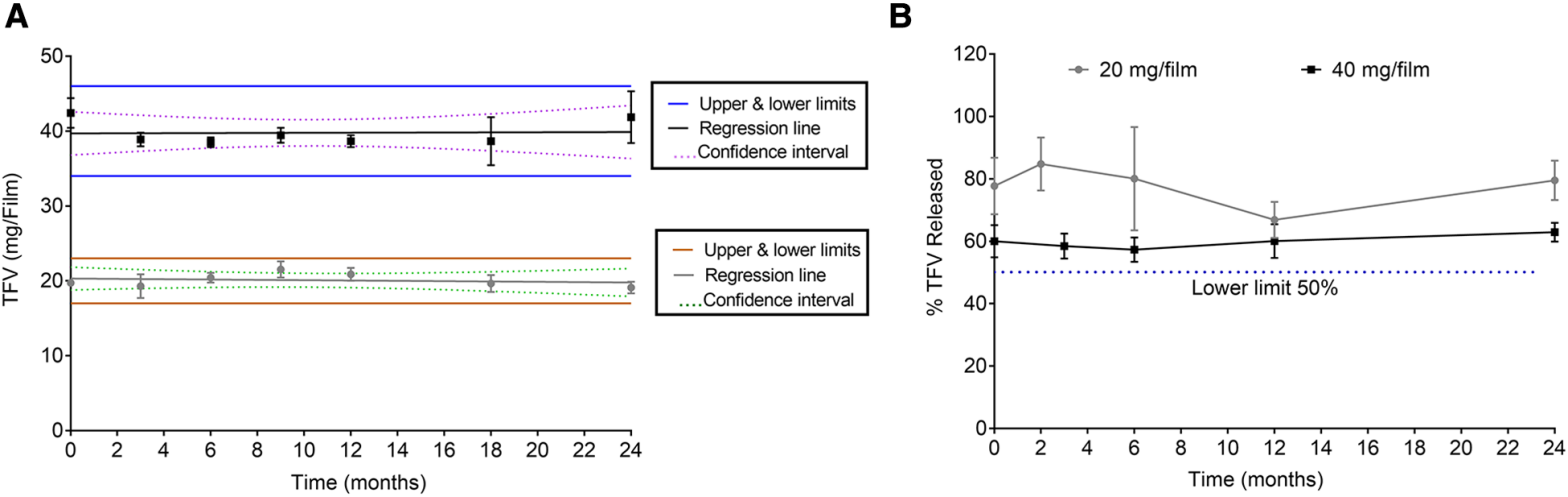
Stability assessment of 20 and 40 mg films at 25°C/60% RH storage conditions. (**A**) Drug content (*n* = 5). (**B**) Percent dissolution at 15 min (*n* = 3). Drug content from day zero was used for percent dissolution calculations.

To determine that films remain stable during storage, stability of low and high dose TFV films was monitored at room temperature (25°C/60% RH) and accelerated (40°C/75% RH) storage conditions as per ICH guidelines. Weight and thickness of the films remained unchanged throughout the stability testing period. As shown in Figure 4 and Supplementary Figure S3, drug content remained within the acceptable limits (85%–115%) for low and high dose films for 24 months at long-term storage and 6 months at accelerated storage conditions. At all the time points evaluated, the percent drug release in the *in vitro* dissolution method was greater than 50% at the 15 min dissolution time point (Figure 4B and Supplementary Figure S3). Puncture strength remained within the acceptance criteria at different time points and conditions tested (data not shown). Water content remained below 10% w/w at all time points tested. TFV films showed compatibility with various strains of lactobacilli throughout the stability testing period (Supplementary Tables S2–S5).

### 3.2. Film evaluation in pigtail macaques

#### 3.2.1. Safety assessment

The safety of the TFV film products (5.1 and 11.2 mg/1.1″ × 1.1″ film) was evaluated in a multiple exposure setting over a two-week period. Table 1 shows the schedule for product administration and biological sample collection. The goal of the safety study was to determine if daily use of either placebo or TFV-containing films show any safety-related changes within the female genital tract and whether these effects are attributable to film formulation and/or dependent on TFV dose.

**Figure 5:**
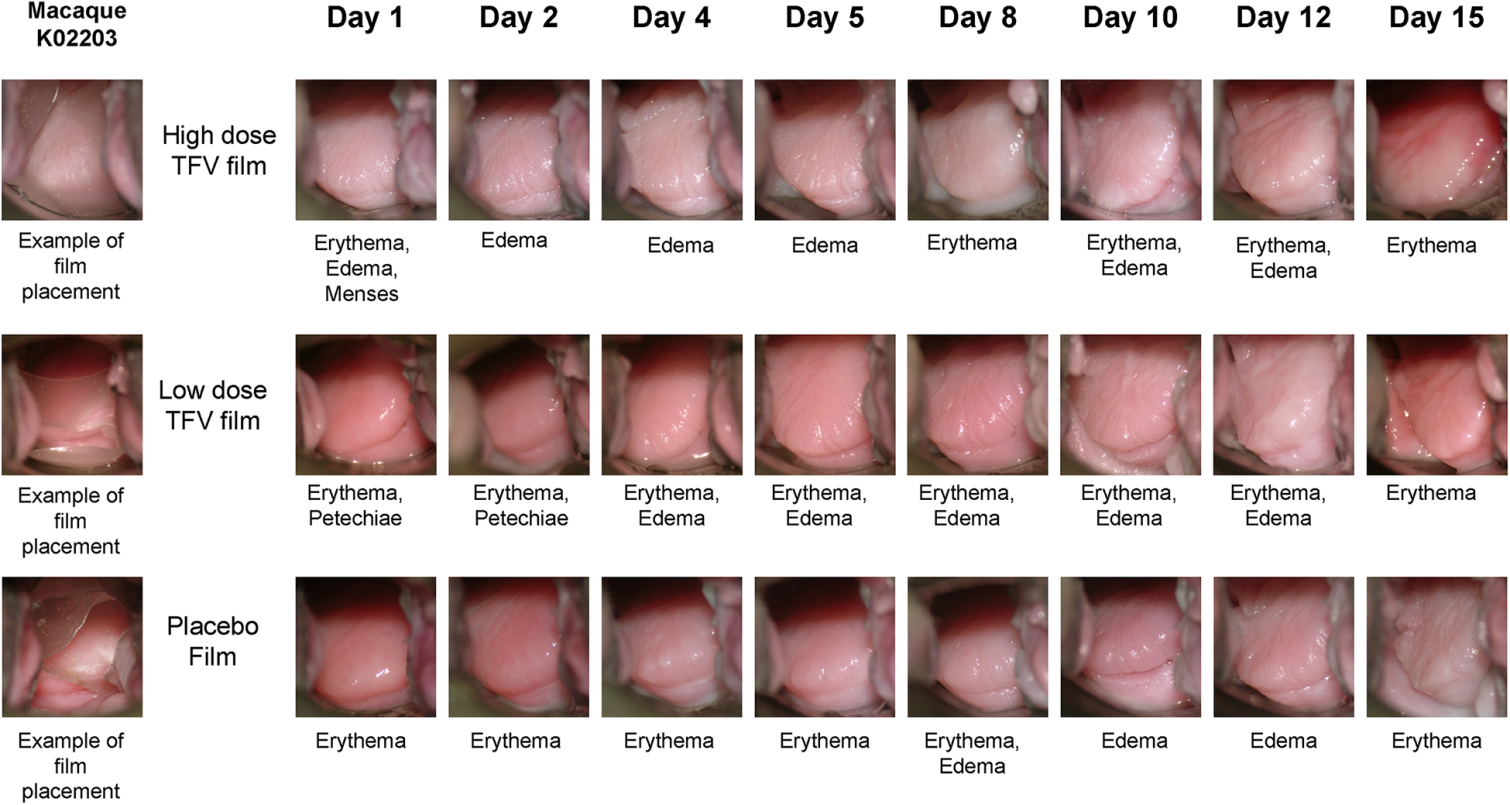
Representative colposcopic images of macaque genital tract showing film placement (left) and observations of adverse events. Adverse events are scored based on visual observation of anatomical changes using our previously published guide (35). Note that the events seen may not be fully captured in the field of view of colposcopic photo.

Safety was assessed using a suite of qualitative (visual) and quantitative assessments. Colposcopy was used to visualize film placement and monitor adverse events based on anatomical and physiological changes (Figure 5). Colposcopic observations commonly noted in the genital mucosal tissues included erythema, edema, petechiae, and grossly white findings (vaginal). Examples of adverse tissue findings included severe erythema, breach in the integrity of the mucosal epithelium and/or tissue friability. Two individual instances of friable areas on a vaginal wall were noted in this study (a single incident in each of the two test product arms, in the same animal). The low incidence and transitory nature of these findings do not indicate a product-related safety concern. Throughout the study, there was no evidence of TFV film related tissue abnormalities.

**Figure 6:**
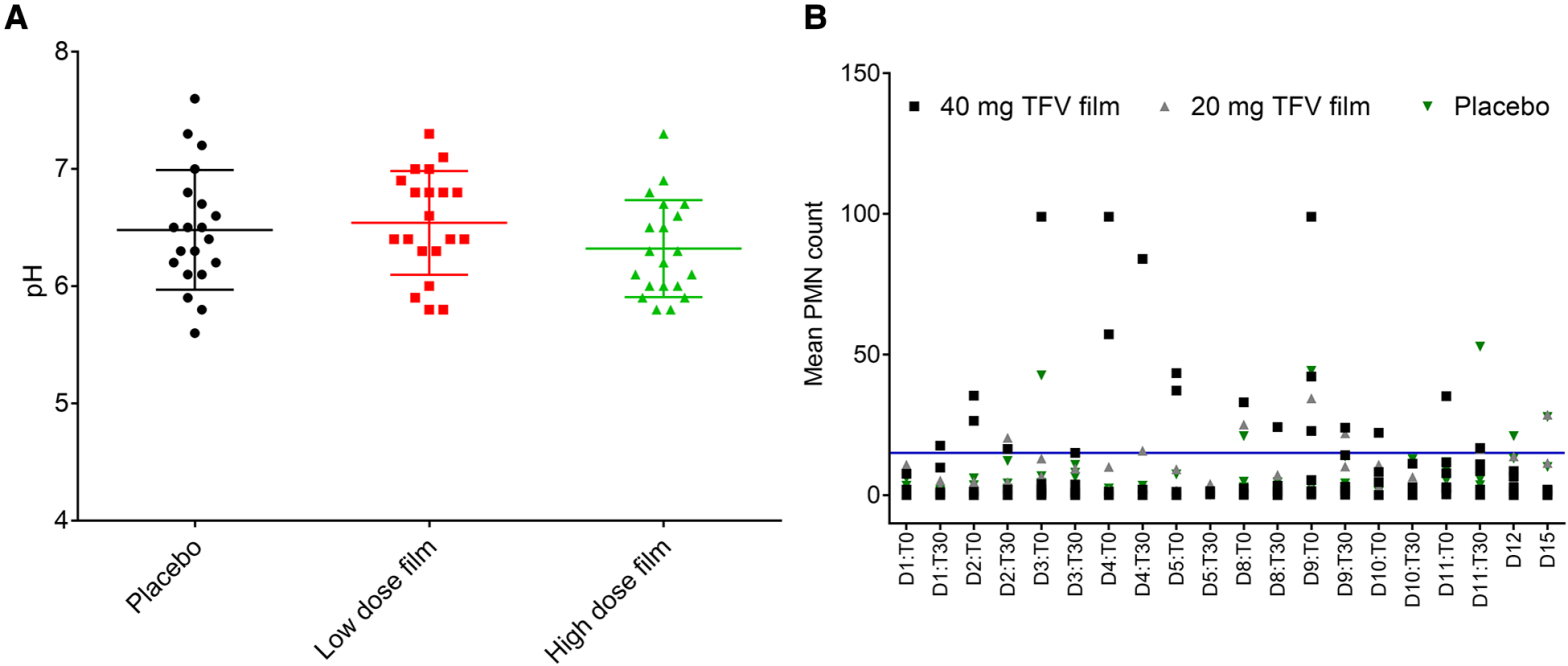
Effect of placebo and TFV (20 mg and 40 mg) films on pH and polymorphonuclear (PMN) cell influx in the female genital tract of macaques. (**A**) Dot plot data shows pH measured for 15 days in macaque cervicovaginal swabs before and after product application. (**B**) Influx of PMNs quantified in vaginal swabs before (T0) and 30 min after (T30) film application and days 12 (D12) and 15 (D15) follow up time period. A transient increase, especially in TFV 40 mg film, was observed, which later receded to around baseline levels in the follow up period. Blue line shows the upper limit of normal range (0–15) of PMN presence in pigtail macaques (unpublished data).

Vaginal pH did not differ by the type of product or length of use. As shown in Figure 6A, pH remained within 5.5 and 7.6 during the two-week study period and no statistical differences were observed between the three arms. Following TFV film exposure, mean polymorphonuclear (PMN) counts increased notably in the higher dose TFV film arm, and remained within the normal range in the lower dose TFV and placebo arms (Figure 6B). The increase observed in the high dose film was sporadic. However, by the end of the study and in the follow-up period (Days 12 and 15), the mean PMN counts subsided to the normal range. Product impact on vaginal microbiota was determined by increase or decrease in growth of selected microorganisms over time. Product use did not impact colonization by lactobacilli and viridians streptococci which produce H_2_O_2_. Apart from small transient changes at few time points, in general, no adverse changes were noted for either of these microorganisms. Overgrowth of deleterious populations of microorganisms such as *E. coli* and *S. aureus* was monitored. Small changes were noted in *E. coli* and *S. aureus*, but they did not coincide specifically with product insertion days. *G. vaginalis* was not detected in any of the macaque study arms. Fluctuations in the presence of some other microorganisms were noted across all three study arms, notably the non-H_2_O_2_ producing lactobacilli and viridans, and aerobic gram-positive rods and cocci. The significance of these shifts is unknown and not deemed significant to the safety profile.

#### 3.2.2. Pharmacokinetic evaluation of TFV film vs. gel

**Figure 7:**
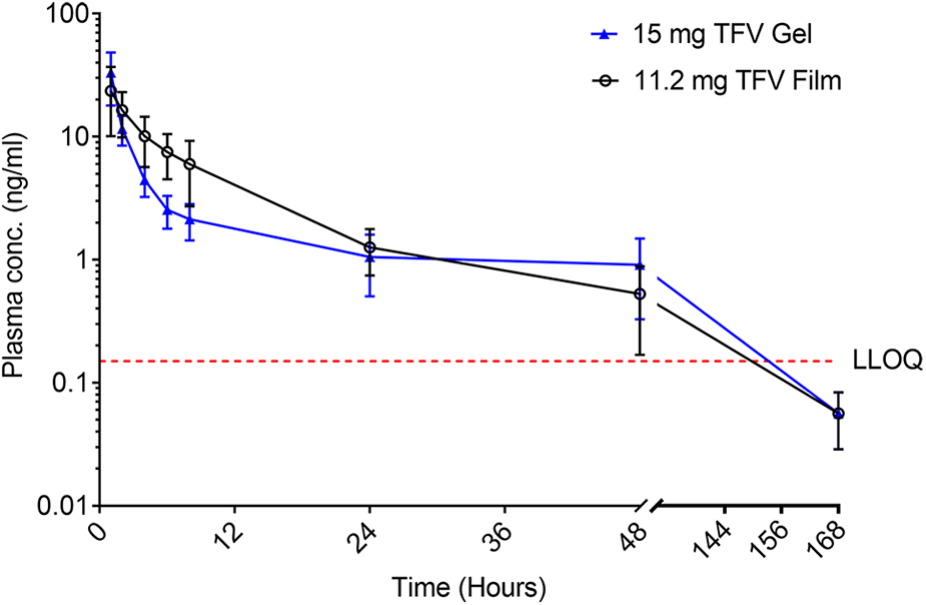
Plasma concentration (mean ± SEM) of TFV vs. time of TFV film and gel products administered vaginally in pigtail macaques. LLOQ–lower limit of quantitation.

Plasma TFV levels reached peak concentrations between 1 h (gel) and 4 h (Film) before decaying gradually over time to concentrations below the lower limit of TFV quantitation between 7 and 24 h post-dosing (Figure 7 and Table 4). The point estimates for all reported PK parameters were numerically higher for the 11.2 mg film when compared to the gel, however, testing for paired differences, no statistical significance was noted.

**Table 4:** Non-compartmental pharmacokinetic parameter estimates for plasma and observed sample times for tissue. Values are median (lower quartile, upper quartile).

Matrix-Analyte	Parameter	Units	Film 11.2 mg	Gel 15 mg
Plasma TFV	T_max_	hrs	4 (1, 8)	1 (1, 2)
	C_max_	ng/ml	20.4 (5.6, 60.1)	17.9 (8.0, 66.5)
	C_max_/D	ng/ml/mg	1.8 (0.5, 5.4)	1.2 (0.5, 4.4)
	T_last_	hrs	24 (8, 72)	7 (2, 17)
	AUC_last_	ng-hr/ml	189 (132, 333)	94 (44, 243)
	AUC_last_/D	ng-hr/ml/mg	16.9 (11.8, 29.8)	6.3 (2.9, 16.2)
Vaginal Tissue TFV	24-hr	ng/mg	11.5 (2.8, 197.6)*	0.4 (0.2, 3.2)
	168-hr	ng/mg	0.10 (0.07, 0.12)	BLQ (BLQ, 0.08)
Vaginal Tissue TFV-DP	24-hr	fmol/mg	274 (76, 1,860)^#^	4 (BLQ, 58)
	168-hr	fmol/mg	BLQ (BLQ, BLQ)	BLQ (BLQ, BLQ)

T_max_, time to peak concentration; C_max_, peak concentration; C_max_/D, C_max_ divided by dose in mg; T_last_, time to last concentration above the LLOQ; AUC_last_, area under the concentration time curve to the last concentration; AUC_last_/D, AUC divided by dose in mg.

BLQ, below the lower limit of assay quantitation (LLOQ); LLOQ plasma TFV 0.31 ng/ml, tissue (median of LLOQ for each sample times biopsy weight) TFV 0.003 ng/mg, TFV-DP 3.5 fmol/mg.

* *p* = 0.016 Film 11.2 mg vs. Gel 15 mg.

^#^ *p* = 0.055 Film 11.2 mg vs. Gel 15 mg.

TFV concentrations in vaginal tissue 24 h after dosing were measurable in all animals in both arms. The concentrations following the 11.2 mg film were greater than those of the 15 mg gel (*p* = 0.016); by 168 h, only the 11.2 mg film formulation had quantifiable TFV concentrations in most animals (Table 4). At 24 h, TFV-DP was quantifiable in all animals receiving the 11.2 mg film and approached statistical significance (*p* = 0.055) compared to gel arm, which had quantifiable concentrations in one-half or fewer of the animals; by 168 h, all, except one, TFV-DP samples were below the LLOQ.

## 4. Discussion

Given the favorable acceptability and ease of administration, thin films are an advantageous dosage form for vaginal delivery of PrEP against HIV and HSV-2. We postulated that the film dosage form can be utilized for delivery of TFV at a dose similar to that previously utilized in the clinically evaluated TFV gel formulation, which amounted to 40 mg of TFV per film. We describe here the development of a thin polymeric film carrying low (20 mg) and high (40 mg) dose of TFV, and evaluation for PK and safety in pigtail macaques. The goal of this work was to provide an alternate TFV vaginal product to a gel that eliminates some of the barriers for acceptable user qualities while maintaining adequate characteristics, performance, and safety. Further, vaginally applied films may have enhanced user acceptability due to advantages including portability, avoidance of applicator for administration that impact cost, ease of use, discretion and lack of leakage.

Film formulation development efforts were met with several challenges related to TFV solubility in film excipients and physical stability. The choice of major excipients was limited due to their impact on film quality and inability to form a continuous matrix required for film formation. The use of solubilizers such as surfactants and cosolvents to achieve target TFV dose was intentionally avoided in this work due to published reports suggesting potential for an increase in viral infectivity in vaginal tissue with commonly used solubilizing excipients (40). Initial studies attempted to identify a polymer matrix that achieved high TFV solubility and remained amenable for convenient manufacturing. Extensive excipient screening by microscopy and visual determination for color change guided selection of polymers that were compatible with TFV. Excipients including HPMCE5, HEC, PVPK90 and NaCMC-LV were selected as film forming polymers due to their acceptable regulatory status, well documented safety profile, and history of use in thin film formulations for vaginal and oral delivery (41–43). An accelerated study at 40°C/75% RH for 7 days (Supplementary Figure S1) suggested that the individual polymer-TFV ratio must be adjusted to 3 or higher to achieve physical stability in any of these individual polymers except HPMCE5. At all polymer concentrations tested, TFV was found to be immiscible with HPMCE5, resulting in phase separation. Given the favorable results from HEC, NaCMC-LV, and PVPK90, a series of prototype placebo formulations were developed using a combination of one film forming polymer (i.e., HEC or PVPK90) and the viscosity enhancer NaCMC-LV. It was identified that greater than 2% w/v NaCMC-LV concentration increased viscosity of the liquid blend drastically leading to bubble entrapment and difficulty in casting films. Therefore, the concentration of NaCMC-LV was maintained at 2% w/v.

Despite the fact that HPMCE5 was immiscible with TFV, it improved the visual and tactile (e.g., flexibility) quality of the films. HPMCE5 addition to the formulation was supported by the hypothesis that the contribution of potential physical instability by HPMCE5 could be mitigated with the addition of other polymers to solubilize TFV. Translucent, yet uniform, films with different amounts of HPMCE5 were manufactured. XRD was used to examine the crystallinity of those samples after subjecting them to 40°C/75% RH for 7 days. As shown in Supplementary Figure S2, no peaks supporting the crystallinity of TFV could be detected in all samples. Although PVPK90 films also inhibited TFV crystallization, the processability of these films was inadequate (hard to peel off the substrate). Therefore, HEC-based formulation was selected for future studies, which produced acceptable films. Furthermore, previous microbicide trials have utilized HEC-based gels with acceptable safety. To increase flexibility of the films, glycerin was incorporated at 2% w/v in the solution. Glycerin also acts as a humectant to preserve water in the films and imparts a smooth and soft feel to the films.

TFV films prepared at low (20 mg) and high (40 mg) doses using the optimized formulation showed acceptable physicochemical attributes. Films had appropriate dissolution, toxicity, and activity results (Figure 2). It is anticipated that the product would be administered close to the time of coitus making rapid drug release optimal to achieve pharmacologically relevant concentrations in the female genital tract. The *in vitro* toxicity assessment in TZM-bl cells and different strains of lactobacilli as well as *ex vivo* tissue viability showed favorable safety results for advancement to preclinical studies (Figures 2, 3). Moreover, TFV films retained antiviral activity in *ex vivo* ectocervical tissues suggesting that TFV exposure from films retained the biological activity and will provide *in vivo* activity.

Both low and high dose TFV films showed excellent stability for 24 months at long-term storage, and 6 months at accelerated storage conditions. All the attributes remained within the specifications and films retained their physicochemical and safety properties supporting further development of TFV films as a marketable product. TFV films produced in this work remained soft, smooth and flexible throughout the stability testing period (Figure 4 and Supplementary Figure S3). Overall, the developed film platform was shown to be safe and stable.

Based on favorable physical properties and *in vitro*/*ex vivo* toxicity profile, both the low and high dose TFV films were advanced to preclinical animal testing in a well-established pigtail macaque model. The safety profile of the developed TFV film was evaluated after repeated film exposure (Table 1) by monitoring for changes in pH, PMN infiltration, microbiota, and colposcopy-assisted adverse events. The effect of repeated exposure was investigated for two reasons, firstly to stress the cervicovaginal environment, and secondly to simulate real-use conditions where women tend to use the film repeatedly in a short period of time. In a span of 15 days study period, the genital tract of pigtail macaques was exposed to nine films. Although minor adverse events were noted, these were not related to film use. The vaginal pH was monitored since alteration can impact susceptibility to infections (44). The vaginal pH remained between 5.5 and 7.6 throughout the study, and the effect of TFV product use on pH alteration was not observed (Figure 6). A small rise in PMN infiltration was observed with high dose film during the film exposure period, but subsided to baseline levels at the end of the study. The clinical ramifications of this transient increase in PMNs in the high dose group is not readily discernible. However, this transient increase did not coincide with any tissue-related events from colposcopy and thus does not suggest inflammatory response with product use. Finally, the vaginal microflora, especially the H_2_O_2_-producing lactobacilli remained largely unaltered with product use suggesting the inertness of this platform on innate factors.

While the median for all PK parameters was higher for the higher dose 11.2 mg film than the gel, these were not statistically significant due to inconsistent trends across formulations within individual animals. The more sparsely sampled tissue indicated higher tissue TFV and TFV-DP concentrations with the film compared to the gel, though most samples were below the LLOQ one week after dosing. These results are expected given the leaky nature of the gel compared to films. Films deliver precise doses to the vagina and their low weight contributes to insignificant leakage and dilution of the innate factors. In two separate clinical studies published previously evaluating TFV films, it was shown that the 40 mg TFV films developed here have shown TFV and TFV-DP levels similar to or higher than gels in vaginal fluids, plasma, and tissues and also suggested increased acceptability among women participants (30, 31). Overall, the TFV high dose film was found to be stable, efficacious, and safe based on *in vitro*, *ex vivo*, and *in vivo* studies. Importantly, high dose film showed TFV and the active metabolite levels similar or better than the gel formulation indicating potential effectiveness against HIV and HSV-2 acquisition in women. Two doses of TFV films (10 mg and 40 mg) were subsequently scaled-up and investigated in healthy women for safety, acceptability, and pharmacokinetics (30, 31).

## 5. Conclusions

A stable vaginal thin film platform that incorporated clinically relevant dose of TFV (40 mg) and non-toxic excipients was successfully developed. TFV was shown to retain antiviral activity *in vitro* and *ex vivo* when formulated into the film dosage form. Safety of the developed film formulation was supported through *ex vivo* exposure studies and *in vivo* studies in the macaque model. TFV films did not show any toxic effects on the vaginal epithelium. The TFV film formulations were shown to retain similar physicochemical characteristics and performance attributes for at least 24 months. In the *in vivo* macaque studies, the high dose TFV film showed higher tissue TFV exposure compared to a gel product. Combined, these findings support advancement of this rationally designed quick-release TFV film product as an on-demand product choice for women at increased risk of HIV and HSV-2 infections.

## Data Availability

The datasets presented in this article are not readily available because the data sets may not be released given the IP restrictions around Film development. Requests to access the datasets should be directed to rohanlc@upmc.edu.
