# Supplementary material for: Tenofovir vaginal film as a potential MPT product against HIV-1 and HSV-2 acquisition: formulation development and preclinical assessment in non-human primates
Source: Front Reprod Health. 2023 Aug 10;5:1217835. doi: 10.3389/frph.2023.1217835 (PMC10449455; doi:10.3389/frph.2023.1217835)
Supplement: Supplementary file 1 [file Datasheet1.pdf]

Supplemental Information

Tenofovir Vaginal Film as a Potential MPT product against HIV-1 and HSV-2 Acquisition: Formulation Development and Preclinical Assessment in Non-human Primates

Methods & Results

Viscosity Determination

HEC or PVPK90 with solution concentration of 6% w/w was mixed with NaCMC-LV with solution concentration of 2%/4%/6% w/w in MilliQ water. The viscosity of the resulting solution was measured by a Brookfield viscometer. Temperature during the measurement was maintained at 25°C and spindle speed used was 15 rpm. Average of three measurements was taken as the final result.

Table S1. Effect of NaCMC-LV on blend viscosity

Formulation	Excipient (%wt. of polymer in the solution)			Viscosity(cp)
	HEC	PVPK90	NaCMC-LV	
F1	6	0	2	918.23(±11.27)
F2	6	0	4	2464.73(±76.26)
F3	6	0	6	5325.29(±247.93)
F4	0	6	2	655.88(±14.91)
F5	0	6	4	2061.99(±22.78)
F6	0	6	6	5281.56(±701.45)

Data presented as mean (± SD)

X-ray diffraction (XRD) of multicomponent samples

TFV was dissolved in MilliQ water using sodium hydroxide equimolar to TFV on a magnetic stirrer. Polymers were then dissolved to achieve required ratio. 500 µL of the resulting was transferred onto a 12-well cell culture plate and dried at 65°C for 3.5 h. The crystallinity of samples containing multiple components were determined by X-ray diffraction. Samples were peeled off the culture plate and put on a glass slide. A Bruker D8 diffractometer with a Cu-Kα source and a lynx eye detector was used. Samples were scanned for 9 minutes between 5°-50° (two theta) with a step size of 0.04° and scan step time of 0.5 sec.

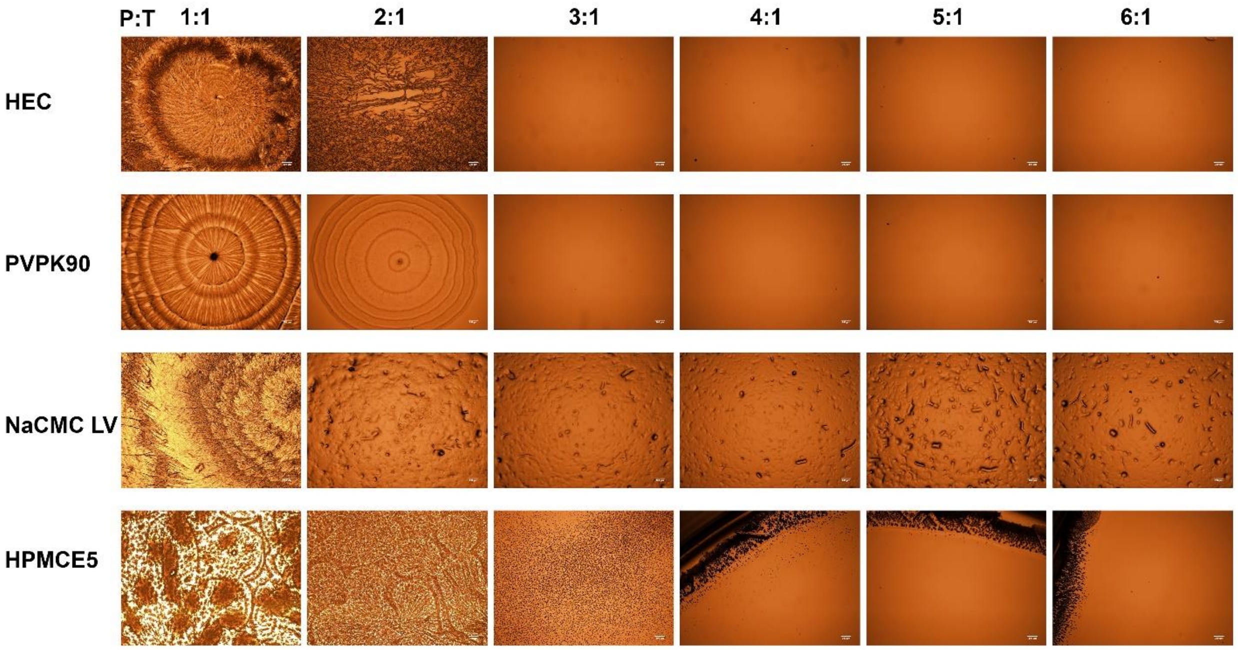

Figure S1. Microscope images of polymer:TFV at different ratios. Scale Bar:200µm.

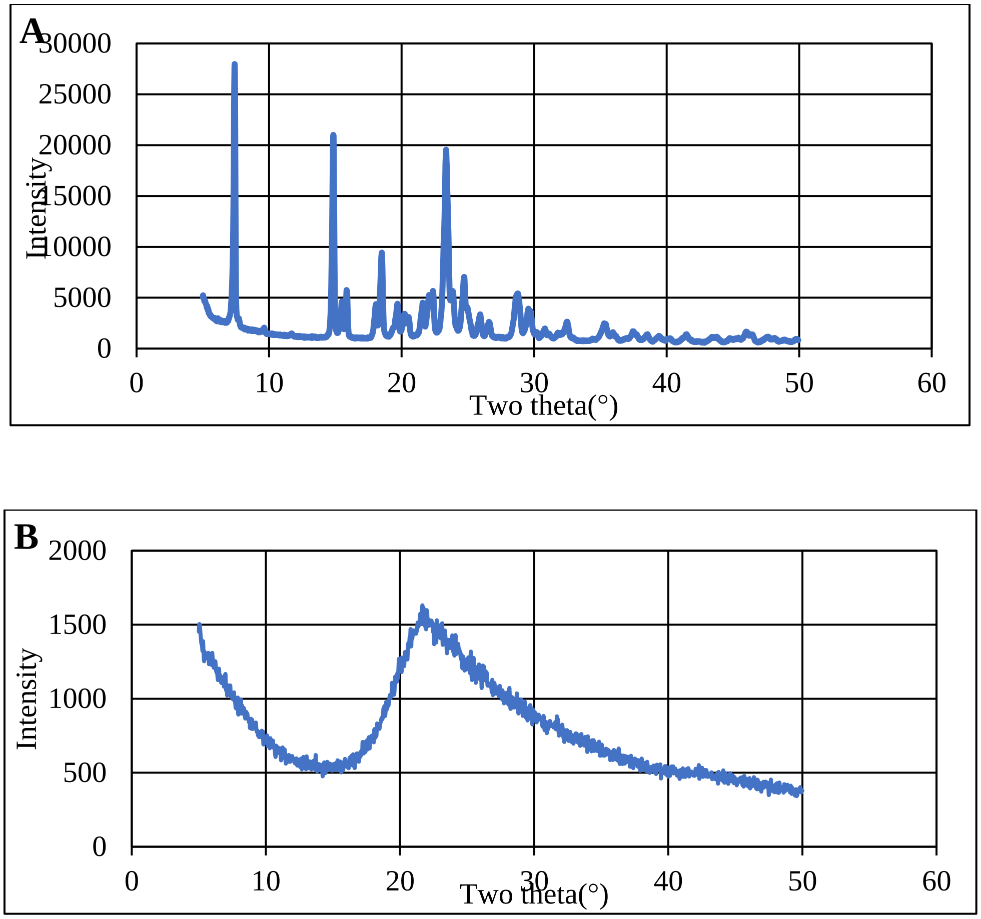

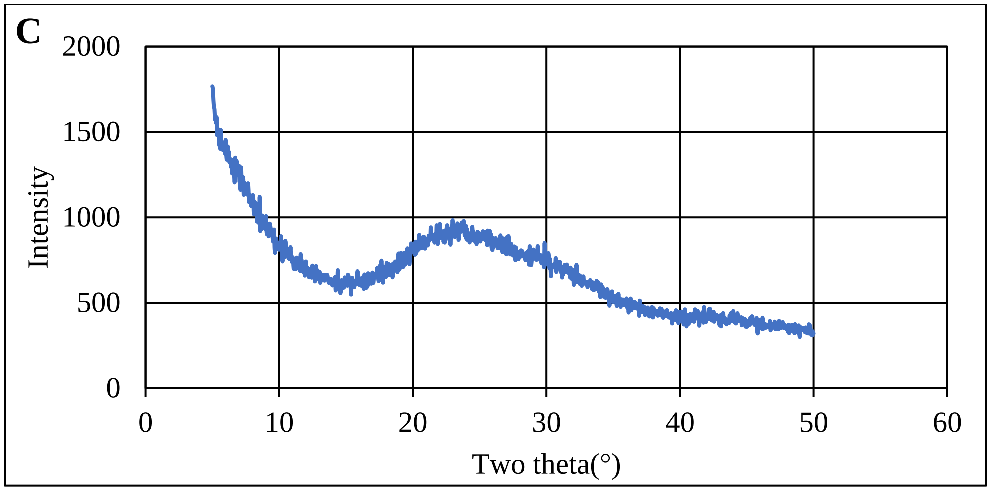

Figure S2. XRD patterns of multicomponent samples. A. Pure Tenofovir. **B.** THCE-1313 represented the ratio of TFV/HEC/CMCNa-LV/HPMCE5 was 1/3/1/3. **C.** TPCE 1313 represented the ratio of TFV/PVPK90/CMCNa-LV/HPMCE5 was 1/3/1/3.

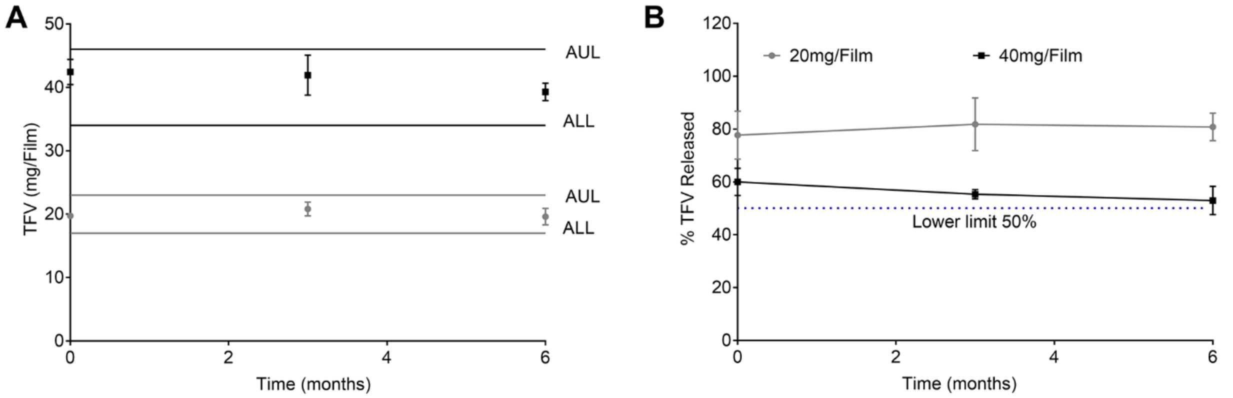

Figure S3. Film characterization under accelerated storage conditions (40 °C/75% RH) for 6 months. **A.** Assay **B.** % dissolution at 15 min**.**

Table S2. Effect of 20 mg tenofovir film on lactobacilli viability during stability storage (25°C/60%RH)

**Strain**	**Time 0**	**3 month**	**6 month**	**18 month**	**24 month**
L. crispatus ATCC 33197	-0.047	0.177	0.092	0.110	0.198
L. jensenii ATCC 25258	-0.005	-0.016	-0.196	-0.302	-0.063
L. jensenii LBP 28Ab	0.054	-0.090	-0.185	-0.097	-0.264

Table S3. Effect of 20 mg tenofovir film on lactobacilli viability during stability storage (40°C/75%RH)

Strain	Time 0	3 month	6 month
L. crispatus ATCC 33197	-0.047	-0.007	-0.066
L. jensenii ATCC 25258	-0.005	0.074	-0.103
L. jensenii LBP 28Ab	0.054	-0.191	-0.152

Table S4. Effect of 40 mg tenofovir film on lactobacilli viability during stability storage (25°C/60%RH)

Strain	Time 0	3 month	6 month	18 month	24 month
L. crispatus ATCC 33197	-0.144	-0.006	0.005	-0.045	0.140
L. jensenii ATCC 25258	0.004	-0.004	0.000	-0.270	0.049
L. jensenii LBP 28Ab	0.115	-0.138	0.035	-0.271	-0.007

Table S5. Effect of 40 mg tenofovir film on lactobacilli viability during stability storage (40°C/75%RH)

Strain	Time 0	3 month	6 month
L. crispatus ATCC 33197	-0.144	-0.053	0.048
L. jensenii ATCC 25258	0.004	0.047	-0.122
L. jensenii LBP 28Ab	0.115	-0.016	0.033
